# Behavior of dicentric chromosomes in budding yeast

**DOI:** 10.1371/journal.pgen.1009442

**Published:** 2021-03-18

**Authors:** Diana Cook, Sarah Long, John Stanton, Patrick Cusick, Colleen Lawrimore, Elaine Yeh, Sarah Grant, Kerry Bloom

**Affiliations:** Department of Biology, The University of North Carolina at Chapel Hill, Chapel Hill, North Carolina, United States of America; Duke University, UNITED STATES

## Abstract

DNA double-strand breaks arise *in vivo* when a dicentric chromosome (two centromeres on one chromosome) goes through mitosis with the two centromeres attached to opposite spindle pole bodies. Repair of the DSBs generates phenotypic diversity due to the range of monocentric derivative chromosomes that arise. To explore whether DSBs may be differentially repaired as a function of their spatial position in the chromosome, we have examined the structure of monocentric derivative chromosomes from cells containing a suite of dicentric chromosomes in which the distance between the two centromeres ranges from 6.5 kb to 57.7 kb. Two major classes of repair products, homology-based (homologous recombination (HR) and single-strand annealing (SSA)) and end-joining (non-homologous (NHEJ) and micro-homology mediated (MMEJ)) were identified. The distribution of repair products varies as a function of distance between the two centromeres. Genetic dependencies on double strand break repair (Rad52), DNA ligase (Lif1), and S phase checkpoint (Mrc1) are indicative of distinct repair pathway choices for DNA breaks in the pericentromeric chromatin versus the arms.

## Author summary

A challenge in chromosome biology is to integrate the linear code with spatial organization and chromosome dynamics within the nucleus. The major sub-division of function in the nucleus is the nucleolus, the site of ribosomal RNA synthesis. We report that the pericentromere DNA surrounding the centromere is another region of confined biochemistry. We have found that chromosome breaks between two centromeres that both lie within the pericentromeric region of the chromosomes are repaired via pathways that do not rely on sequence homology (MMEJ or NHEJ). Chromosome breaks in dicentric chromosomes whose centromeres are separated by > 20 kb are repaired via pathways that rely mainly on sequence homology (HR, SSA). The repair of breaks in the pericentromere versus breaks in the arms are differentially dependent on Rad52, Lif1, and Mrc1, further indicative of spatial control over DNA repair pathways.

## Introduction

Chromosomes containing two functional centromeres (dicentrics) are subject to a breakage-fusion-bridge (BFB) cycle as cells divide [1–4]. The BFB cycle is a hallmark of genetic instability that promotes oncogenesis. Using a conditionally functional centromere (transcriptional promoter GAL1 adjacent to eCEN3, GALCEN3), we can regulate onset of the BFB cycle in order to examine breakage and subsequent DNA repair pathways [5,6]. In cells with a dicentric chromosome, there is an approximately 50/50 chance that centromeres on the same DNA strand will go to the same or opposite spindle pole during mitosis [7]. When centromeres on the same chromatid strand go to the same pole, sister chromatids segregate without DNA breakage. Chromosome breakage, and the resulting BFB cycle, ensues when centromeres on the same strand orient to opposite poles [7]. Forces that sever the DNA come from cell wall closure following cytokinesis [8,9]. About 50% of the DNA breaks occur within 10 kb of either of the two centromeres [10,11].

Budding yeast utilize a variety of DNA repair pathways to resolve to monocentric chromosomes following dicentric chromosome breakage. These include homology-based pathways (HR and SSA)[6,7,12], telomere addition to broken ends [13], end-joining via non-homologous (NHEJ) or micro-homology mediated processes (MMEJ)[10], and break-induced replication (BIR)[9]. Homology-based pathways (HR and SSA) are initiated through resection of 5’ ends to allow invasion of a single-strands into regions of intra- or interchromosomal homology. In the case of dicentric chromosomes with non-homologous or inverted homologous centromeres, repeated TY elements serve as sites for homology-based repair [6,14]. Dicentric chromosomes containing homologous centromeres in a direct orientation can repair through single-strand annealing (SSA) between the two centromeres [5,7], generating a linear monocentric deletion derivative chromosome. Cells containing dicentric chromosomes in which the centromeres are 46.3 kb from one another that lack *rad52Δ* or *rad1Δ* are inviable (< 2.0%), diagnostic of homology-based repair such as SSA [12]. In the absence of NHEJ (Ku or Sir2) cell viability is reduced by about 50% [10,15,16], indicative of the contribution, albeit to a lesser extent, of non-homologous end-joining repair events. In contrast, the efficiency of transformation of dicentric plasmids (~11–15 kb) [17] into strains lacking end-joining proteins Ku, Sir2, 3, and 4, is reduced 20-30X [18]. The range of viability of cells containing dicentric chromosomes or plasmids in Ku or Sir2 mutant backgrounds could reflect differences in the topology of circular vs. linear chromosomes, the distance between the two centromeres, or other mechanisms that might bias the choice of repair pathway.

The 16 centromeres in budding yeast are physically clustered for the majority of the cell cycle due to their persistent attachment to kinetochore microtubules [19,20]. Their proximity may influence repair pathways following DNA damage. In addition, different sub-nuclear domains, such as pericentromere vs. centromere [21,22], nucleolus vs. nucleus [23], and heterochromatin vs. euchromatin may not only define transcriptional domains but could also dictate repair pathway choice following DNA damage. The nucleolus, which is comprised of ~9.1 kb ribosomal DNA (rDNA) repeats, repairs lesions using intrachromatid recombination, resulting in the production of extrachromosomal rDNA circles [24]. DSBs in the rDNA repaired via HR make excursions outside the nucleolus in order to gain access to the homology-based recombination machinery [25]. The pericentromere has several features in common with the nucleolus, such as increased concentration of the SMC proteins condensin and cohesin and a high prevalence of replication fork stalling [19,26,27]. It is unknown how the unique environment of the pericentromere may influence repair requirements between yeast centromeres with 125 bp of homology. The repair mechanisms within the pericentromere may reveal unique biochemical attributes of this domain.

The pericentromere in budding yeast is streamlined in terms of repeat sequences that are characteristic of organisms with regional centromeres (e.g. mammals). The challenge in systems with a high number of alpha satellite repeats is the potential for loss or gain of DNA repeats due to incorrect duplication or repair. One strategy is to suppress the ATR checkpoint in order to prevent checkpoint activation upon replication fork pausing [28]. An alternative strategy is the activation of a non-canonical ATR pathway that prevents the formation of lagging chromosomes [29]. In budding yeast, checkpoint proteins Mrc1, Tof1, and Csm3 localize to replication forks [30], with distinct roles at the centromere [31]. These unique aspects of regional centromeres warrant investigation of repair pathways that have evolved to prevent the shuffling or loss of alpha satellite DNA repeats, which are essential to the faithful segregation of chromosomes.

We used a series of dicentric chromosomes separated from 6.5 kb to 57.7 kb to explore the possibility that local chromosomal domains or the position of the DSB could influence repair pathway preference. We found that end-joining is the favored repair pathway when the two centromeres are less than 20 kb apart, within the pericentromere, while homology-based pathways (SSA) become the dominant repair pathway for centromeres much greater than 20 kb from one another.

## Results

### Viability of cells experiencing dicentric chromosome breakage is dependent on location of the GALCEN

**Fig 1:**
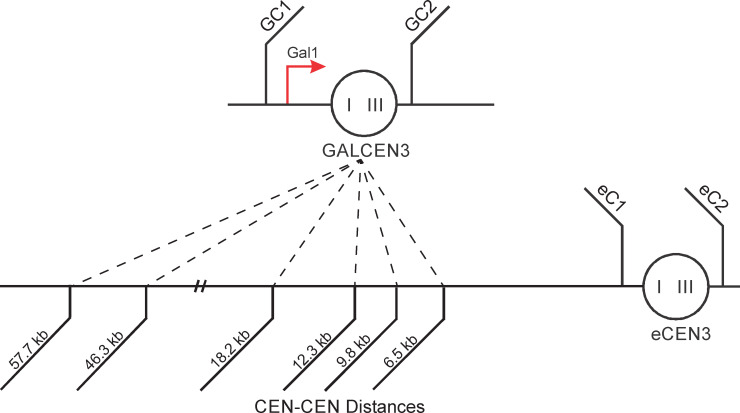
Dicentric Chromosome III. A schematic depicting the conditional GALCEN3 centromere, which is inactive on galactose and active on glucose. The locations of the GALCEN3 insert on Chr. III relative to the endogenous CEN3 are also indicated. Primers used to probe for the presence of the intact centromere are shown. GALCEN3 was inserted 6.5 kb (107,830 bp in Chromosome III), 9.8 kb (104,458 bp), 12.3 kb (101,976 bp), 18.2 kb (96,020 bp), and 46.3 kb (68,000 bp at *HIS4*) and 57.7 kb (56,740 bp) from the endogenous centromere eCEN3 (114,300); TCLWTy2-1 spans base pairs 84811–90769.

**Fig 2:**
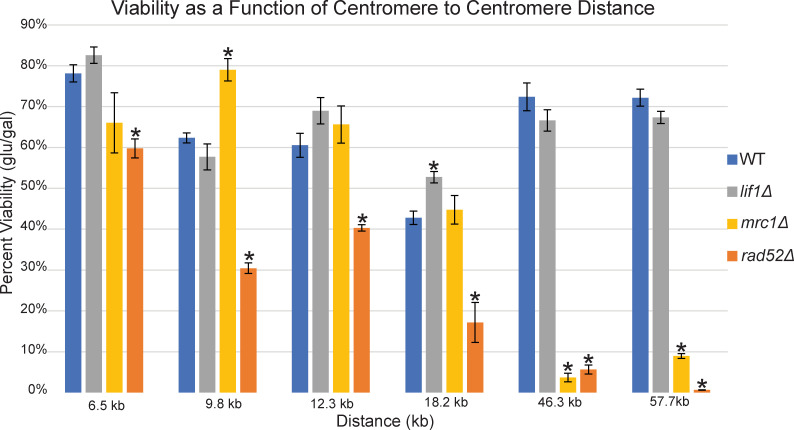
Dicentric Cell Viability. Quantitative analysis of cell viability (single colony growth on glucose/galactose), for strains with GALCEN3 inserted 6.5 kb, 9.8 kb, 12.3 kb, 18.2 kb, 46.3 kb and 57.7 kb from endogenous CEN3. WT, *lif1Δ*, *mrc1Δ*, and *rad52Δ* mutants are shown. Comparing each mutant to its corresponding WT at a given distance, student’s T-test values of <0.05 are marked with an asterisk. Error bars indicate ± SEM. Viability in *rad52Δ* and *mrc1Δ* decreases as the distance between centromeres increases, while *lif1Δ* has no detrimental effect on viability. Each strain was plated at least 3 times. Student’s t-test values can be found in S2 Table.

We used the transcriptionally regulated centromere (GALCEN3) to build conditionally functional dicentric chromosomes with the two centromeres ranging from 6.5 to 57.7 kb apart on chromosome III. GALCEN3 was inserted 6.5 kb (107,830 bp in Chromosome III), 9.8 kb (104,458 bp), 12.3 kb (101,976 bp), 18.2 kb (96,020 bp), 46.3 kb (68,000 bp at *HIS4*) and 57.7 kb (56,740 bp) from the endogenous centromere eCEN3 (114,300) (Fig 1). Cells grown on glucose (conditional centromere active) exhibit heterogeneous colony morphology, consistent with the physical instability of dicentric chromosomes and variation in sister chromatid alignment, the timing of breakage, and the number of cycles to repair to a monocentric derivative [5,7,32]. The cells are able to generate viable monocentric derivative chromosomes to various degrees depending on the distance between the two centromeres (Fig 2, WT). There is variation along the chromosome depending on the placement of the conditional centromere, ranging from a low of 42% (18.2 kb) to a high of 78% (6.5 kb). There are essential genes between the 18.2 and 46.3 kb dicentric (*NFS1*) and another between the 46.3 and 57.7 kb dicentric (*RRP7*). Introduction of *NFS1* into a second site in the genome (*TRP1* locus on chromosome IV) had no effect on the viability or distribution of repair products in strains with the second centromere at 46.3 kb (S1 Fig). The difference in viability among the series of dicentric chromosomes is not correlated with proximity of the conditional centromere to an essential gene.

To determine the spectrum of DNA repair processes in the series of dicentric chromosomes, we examined cell viability in cells lacking Rad52 (HR and SSA), Lif1 (NHEJ), or Mrc1 (replication checkpoint). Inactivating the NHEJ pathway in *lif1Δ* strains did not affect the cell’s ability to resolve dicentric breakage as measured by viability. The variation in viability from one position to another observed in the repair proficient strains was maintained in the absence of Lif1 (Fig 2, grey). In contrast, the viability in *rad52Δ* and *mrc1Δ* mutants dropped significantly as a function of increasing distance between the centromeres. In *rad52Δ*, there was a two-fold drop in viability from 6.5–9.8 kb (Fig 2, orange). At 12.3 kb the viability was slightly higher, but declined with increasing distance from eCEN3 at 18.2 kb, 46.3 kb and 57.7 kb to 17%, 5% and 1% viability, respectively. In *mrc1Δ*, the viability of cells containing dicentric chromosomes with less than 20 kb between centromeres was not negatively impacted, while viability of cells with centromeres at 46.3 and 57.7 kb dropped to less than 10% (Fig 2, yellow).

### Repair pathways for dicentric chromosome induced DSB repair

**Fig 3:**
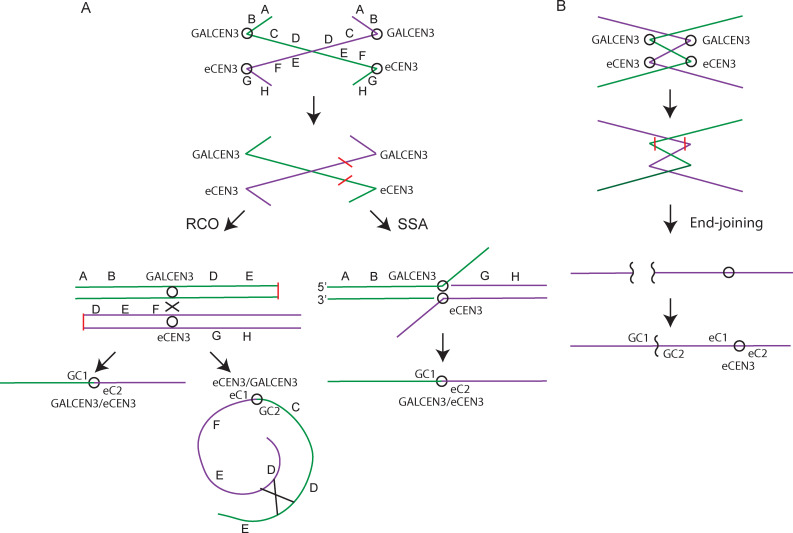
Dicentric Chromosome Repair. Schematic representing sister chromatid alignment, breakage, and repair to monocentric derivatives following activation of the dicentric chromosome. A. Homology-based repair: (RCO) Resolution through a reciprocal crossover between homologous centromeres yields linear and circular monocentric chromosome derivatives. In the linear rearrangement, telomeric ends remain intact resulting in a monocentric deletion chromosome. The reciprocal product contains DNA between the two centromeres (C-D-E-F). An additional event is required for circularization. (SSA) Resolution through single-strand annealing between the left chromosome arm containing GALCEN3 and the right chromosome arm containing eCEN3 yields a linear rearrangement. Telomeric ends remain intact, resulting in a linear monocentric deletion chromosome. ​B. End-joining events lead to a monocentric derivative chromosome where one entire centromere (either GALCEN or eCEN3) is deleted. A deletion of GALCEN3 is depicted (far right, bottom).

To determine the nature of the repair products in the monocentric derivatives, we used PCR to identify major repair products. There are two configurations for biorientation of a dicentric chromosome. Sister kinetochores orient to opposite poles in order to satisfy the spindle assembly checkpoint. The kinetochores at different chromosomal loci behave independently from one another, and thus the outcome is dictated by whether the two non-sister kinetochores on the same strand align to the same or opposite poles. There is an approximately 50% chance that a dicentric chromosome will be broken at each division [5]. When non-sister kinetochores on the same DNA strand align to opposite spindle poles, anaphase bridges arise (Fig 3A) followed by collapse of the mitotic spindle and cell separation. Repair events can lead to monocentric chromosome derivatives or regenerate the dicentric chromosome, which will continue to undergo breakage-fusion-bridge cycles until stable monocentric derivatives are generated [32].

Homology-based mechanisms are the primary pathway for repair of the 46.3 kb dicentric chromosome [6,7,10]. Reciprocal cross-over between the GALCEN3 and eCEN3 results in a linear monocentric derivative chromosome with the centromere fusion (GC1-eC2) and the reciprocal centromere (eC1-GC2). An additional exchange event is required to join the DNA ends of the DNA fragment containing the eC1-GC2 reciprocal centromere to generate the circular derivative (C-D-E-F) containing the precise 46.3 kb of DNA, now absent from the linear monocentric deletion derivative chromosome III (Fig 3A, RCO left panel). A second homology-based mechanism, single-strand annealing between 336 bp of homology between GALCEN3 and eCEN3 (Fig 3A, SSA right panel), generates a linear monocentric derivative chromosome deleted for DNA between GALCEN3 and eCEN3.

We performed DNA sequence analysis for 25 single colonies on glucose to assess gene copy number and determine whether there was loss or gain of information. 17/25 had the full complement of the initial genome, 2/25 were deleted for eCEN3 and 6/25 were deleted for GALCEN3. These data establish the existence of the linear and circular monocentric derivatives arising through centromere DNA homology-based repair mechanisms and subsequent exchange to heal the broken ends (Fig 3). Through nested oligonucleotide pairs, we were able to identify overlapping PCR products and establish 41 kb of contiguous DNA to either side of the reciprocal PCR product containing the centromere (S2 Fig). The remaining 6 kb of the circular product is accounted for by the Ty2-1 element at position 84–90 kb on Chromosome III. We did not distinguish whether these products were generated from a single homologous recombination event or independent SSA events.

Non-homologous events in which either of the two centromeres is deleted are the other observed repair pathway [10]. The non-homologous events preferentially delete GALCEN3 over eCEN3, in about a 70:30 ratio. The entire centromere is deleted and repair ensues using small regions of flanking homology (Fig 3B). The deletions are much larger than predicted from canonical end-joining events, indicative of two breakage events as depicted in Fig 3B.

### Single-strand annealing and non-homologous repair pathways for dicentrics within the pericentromere

To determine the structure of monocentric derivatives in the collection of cells with the two centromeres ranging 6.5 kb to 57.7 kb from one another, we used PCR to identify the un-rearranged centromeres (GALCEN3 and eCEN3) and the hybrid centromeres (GALCEN3/eCEN3 and eCEN3/GALCEN3 shown in Fig 3). As shown in Fig 3 (top), the probability of chromosome breakage is about 50% at each cycle, with repair back to the dicentric state (BFB) as one of the outcomes in the event of a break (depending on resolution of the reciprocal crossover between the two centromeres Fig 3A). We propagated cells 72 hours on glucose followed by plating for single colonies on glucose to minimize the fraction of cells containing intact dicentric chromosomes and ensure single colonies reflect individual repair events.

**Fig 4:**
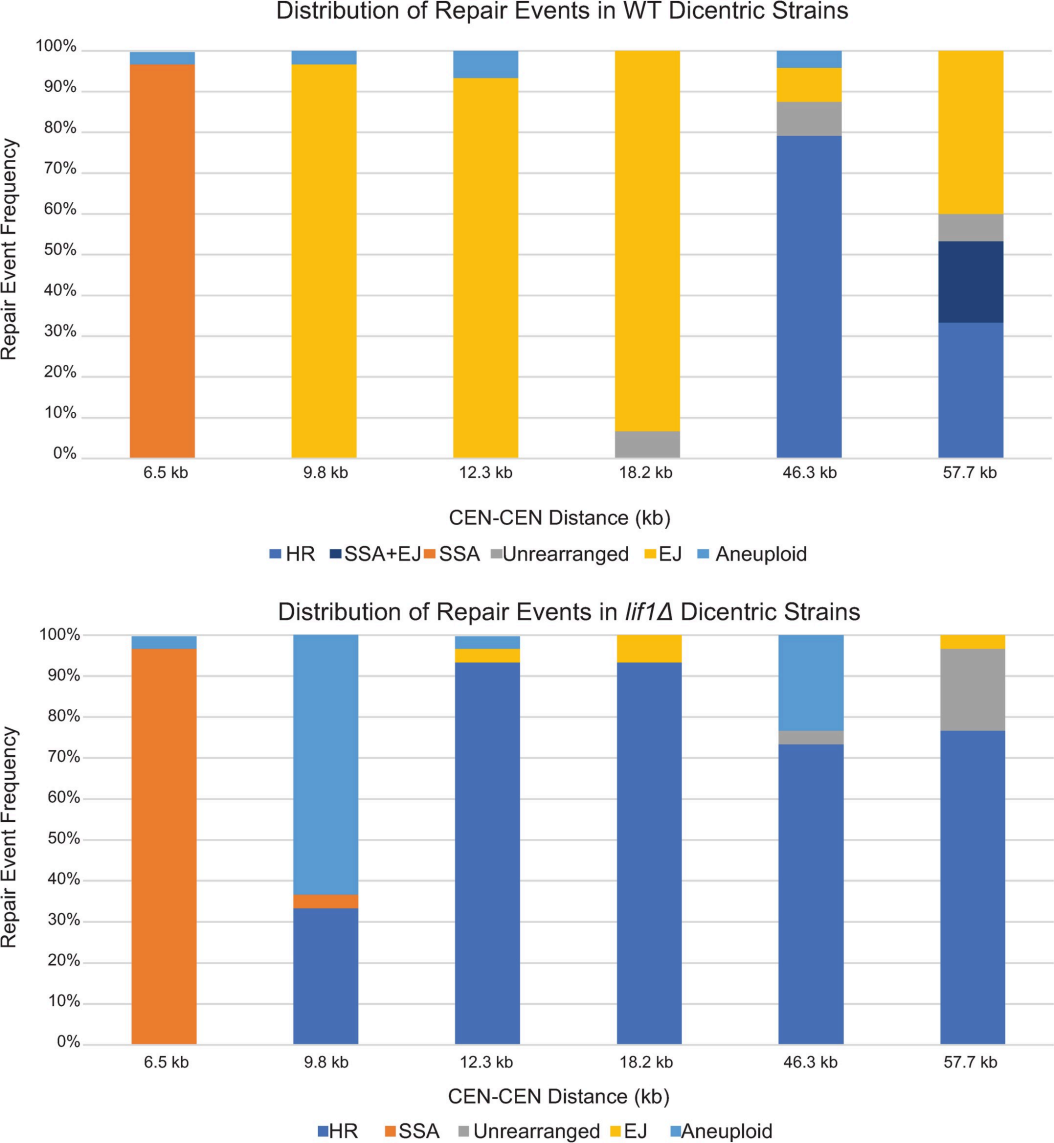
Repair products as a function of dicentric centromere distance. A. Distribution of repair events in dicentric strains with varying CEN-CEN distances. Single colonies were analyzed by PCR that probed for eCEN3 (eC1-eC2), GALCEN3 (GC1-GC2), rearrangement (GC1-eC2), and reciprocal products (eC1-GC2) as shown in Fig 3. At 6.5 kb, SSA is the prominent repair method. At intermediate distances (9.8–18.2 kb), EJ is most common (>90%). At 46.3 kb, HR accounts for repair in 70% of colonies. At 57.7 kb, the events are distributed as follows: 40% EJ, 33% HR, 20% SSA+EJ, 7% unrearranged. Key: HR events were categorized as cells with the rearrangement and reciprocal product (Fig 3) but neither GALCEN3 or eCEN3. SSA events were rearrangement only. Unrearranged meant intact CEN3 and GALCEN3, with no repair products. EJ cells were deleted for one of the two centromeres. Aneuploid events were indicated by the presence of the rearrangement product and one or both of GALCEN3 and eCEN3. SSA+EJ cells had eCEN3 and reciprocal product (Fig 3). 30 colonies were analyzed for each strain. B. Distribution of repair events in *lif1Δ* dicentric strains. At 6.5 kb, the distribution of repair events is not altered. At intermediate distances, EJ is no longer the prominent repair product. The null mutant did not have a detrimental effect on the viability of the strains (see Fig 2). 30 colonies were analyzed for each strain.

97% of the colonies from cells containing the 6.5 kb dicentric chromosome contained the hybrid GALCEN3/eCEN3 product (Fig 4A). The presence of GALCEN3/eCEN3 is predicted from a double-strand break between the two centromeres, followed by resection and reannealing of the 336 bp shared between GALCEN3 and eCEN3 sequences. Such a single-strand annealing event generates a hybrid GALCEN3/eCEN3 resulting in loss of the 6.5 kb of intervening DNA due to cleavage of the 3’ overhang (Fig 3A, right panel).

Examination of the 9.8, 12.3 and 18.2 kb dicentric chromosomes revealed a different pattern of repair products in the monocentric derivatives. In all three of these constructs, the majority of events are non-homologous (>90% Fig 4A). Greater than 90% of single colonies contain one of the parental centromeres and a deletion of the other centromere. The large majority are deletions of GALCEN3 (>90%), with 8–10% deleted for eCEN3. The preferential loss of GALCEN3 is comparable to the bias observed in events from the 46.3 kb dicentric chromosomes (78% loss of GALCEN3, 22% loss of eCEN3, n = 243 [10]) and the bias observed in independent studies of dicentric chromosomes containing GALCEN3 in either chromosome III or two different sites in chromosome V [11].

To address whether there is a bias in growth rate of various deletion derivative chromosomes we examined the growth rates of strains deleted for GALCEN3 (9.8 kb and 46.3 kb EJ GALCEN3Δ), deleted for CEN3 (9.8 kb EJ CEN3Δ), rearranged to linear and circular deletion derivatives (46.3 kb HR) and 6.5 kb deletions following the SSA events observed at the 6.5 kb dicentric (6.5 kb SSA). The strains with centromere deletions via EJ (GALCEN3 or CEN3) or harboring the linear and circular derivatives following HR do not lose any coding information and as such exhibit comparable growth rates (S3 Fig). The growth rates are consistent with the expectation for lack of selection of one event over another. The strain in which SSA was utilized for repair (6.5 kb SSA) exhibits a significant reduction in growth rate (S3 Fig). The SSA event results in loss of the PGS1 gene adjacent to CEN3. The observed slow growth is consistent with previous reports of slow growth in the null mutant [33]. There is no difference in growth rates for cells containing monocentric derivative chromosomes that arise through HR or EJ. There is a strong selection against cells experiencing SSA, in which DNA between the two centromeres is lost.

**Fig 5:**
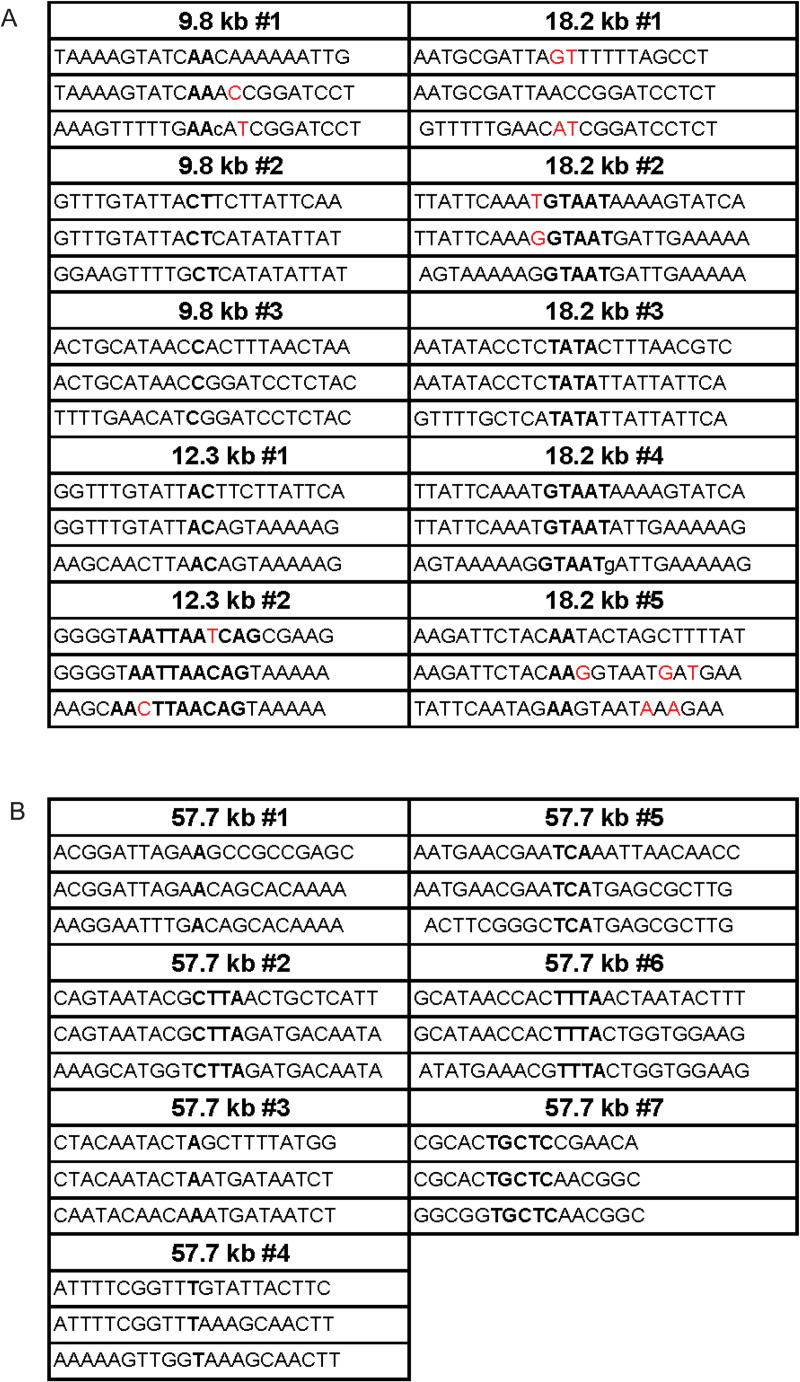
Sequence analysis of GALCEN3 deletions in monocentric derivative chromosomes. GALCEN3 PCR products that were smaller than the wild-type size of 932 bp were sequenced. Top line is normal sequence upstream from GALCEN3, bottom line is normal sequence downstream from GALCEN3, and middle line is the junction found in the repair product. Potential base pairs of homology are bolded. Deletions are lowercase, insertions/mismatches are in red. A. Sequence homology in repair products in pericentric dicentric strains (< 20 kb between centromeres). Junction 18.2 kb #1 had no identifiable homology. B. Sequence homology in repair products in the 57.7 kb dicentric.

To interrogate the mechanism of DSB repair, the junctions of twelve independent isolates of the conditional GALCEN3 deletions were determined by sequence analysis (Fig 5A). The middle line of each represents the actual sequence of the resulting deletion. Above and below this are sequences derived from either side of the junction. Identical bases which may be involved in base pairing during the repair process are highlighted in bold. Deletions are represented in lower case and insertions and mismatches are shown in red. The sequences reveal 1–9 bp of homology at the repair junctions [10]. Based on the length of the homology, these are most likely non-homologous (NHEJ) or micro-homology mediated repair events (MMEJ) [34]. In all cases, there is complete loss of the three centromere DNA elements (CDEI, II, III), with deletions ranging from 278 to 585 bp. The smallest deletion, 278 bp, is similar to the size of the centromere DNA protected from nuclease digestion [35,36]. These findings are consistent with Kramer et al. [10] where 1/5 of the events were deletions <385 bp and 4/5 deletions of >400 bp (n = 198). The large size of the deletions relative to canonical end-joining events (~ 100 bp [37]) and the loss of the entire centromere is indicative of a breakage mechanism that cuts the DNA on both sides of the centromere, such as the cleavage furrow might do at the time of cell abscission (depicted in Fig 3B). This would account for the large size of deletions and the complete removal of all three centromere DNA elements.

Examination of the 46.3 kb and 57.7 kb dicentric chromosomes revealed a third pattern of monocentric derivative chromosomes (Fig 4A). Single cells from the 46.3 kb dicentric chromosome were lacking both parental centromeres, and contained instead two recombinant centromeres, GALCEN3/eCEN3 (GC1/eC2) and eCEN3/GALCEN3 (eC1/GC2). These events could be accounted for by a reciprocal cross-over event, or independent SSA events involving fragments from two different broken chromosomes. One of these events could be annealing between the 336 bp of homology at GALCEN3 and eCEN3, joining fragments containing the left arm of GALCEN3 at HIS4 to eCEN3 and right arm (Fig 3A). A second event could be SSA between 336 bp of homology at GALCEN3 and eCEN3 at the ends of a DNA fragment contiguous from GALCEN3 to eCEN3. Repair of the linear products yields a monocentric circular derivative (Fig 3A, left panel).

At 57.7 kb, resolution was more heterogeneous in terms of pathway choice. One-third of the events were lacking both parental centromeres, and contained two recombinant centromeres similar to the 46.3 kb dicentric (Fig 4A). In about 20% of cells, one chromosome was deleted for GALCEN3, indicative of an end-joining event, and contained one recombinant centromere (eCEN3/GALCEN3), the predicted product of SSA between the ends of a DNA fragment contiguous from GALCEN3 to eCEN3, yielding a monocentric circular derivative. One third of the events were end-joining products in which chromosomes were lacking either GALCEN3 or eCEN3 (Fig 4A). The junctions of 7 independent isolates of the conditional GALCEN3 deletions were determined by sequence analysis (Fig 5B). The sequences reveal 1–4 bp of homology at the repair junctions. As described above, they are most likely non-homologous end-joining events.

### Shifting the spectrum of repair from non-homologous to homology-based repair pathways

The repair pathway in healing dicentric chromosomes could reflect differences in the length of DNA from the break to the region of homology, differential abundance of repair proteins, or different spatial domains of the nucleus. To determine whether the pericentric dicentrics (< 20 kb between centromeres) are accessible to repair via homology-based mechanisms, we examined the spectrum of repair products in the dicentric chromosomes in strains lacking a component of the DNA ligase IV complex, Lif1. Lif1 is a scaffold protein that contributes to the stability and activity of the DNA ligase Dnl4, and together with Dnl4 is also a potent inhibitor of 5’ to 3’ end resection [38]. *lif1Δ* strains containing the suite of dicentric chromosomes from 6.5 to 57.7kb were grown on glucose for 72 hours and plated for single colonies. Single colonies were picked and analyzed for the parental and hybrid centromeres as discussed above. As shown in Fig 4B, the spectrum of repair pathways is dramatically shifted in the pericentric dicentrics. In the case of 12.3 and 18.2 kb, the distribution shifts from 90% non-homology in WT to 90% homology-based repair in *lif1Δ*. In these homology-based repair products, the cells lack both parental centromeres and contain exclusively GALCEN3/eCEN3 (GC1/eC2) and eCEN3/GALCEN3 (eC1/GC2). Thus repair of broken dicentric chromosomes when the centromeres are within 20 kb of each other in the pericentromeric chromatin region can occur via homology-based mechanisms when end joining pathways are disrupted.

DNA repair within the pericentromere is not uniform. In the 9.8 kb dicentric, the fraction of non-homologous repair products drops in *lif1Δ*, but with a different pattern than observed in the 12.3 and 18.2 kb dicentric chromosomes. 35% of colonies exhibit the two hybrid centromere products and lack both unrearranged centromeres. To establish the structure of the predicted circular monocentric derivative, we used nested oligonucleotide pairs to identify overlapping PCR products and mapped 9.8 kb of contiguous DNA to either side of the GALCEN3/eCEN3 hybrid centromere (S5 Fig), similar to that found in the WT 12.3 kb dicentric (S4 Fig). However, 65% of colonies contained two centromeres, one the GALCEN3/eCEN3 rearrangement product and one an unrearranged centromere. We denote these as aneuploid, indicative of multiple events per cell that give rise to two stable centromeres, reflective of two stably segregating chromosomes.

In the arm dicentrics (46.3 and 57.7 kb) lacking *lif1Δ*, there was a decrease in non-homology based products (Fig 4B). For cells containing these dicentric chromosomes, the homology-based mechanisms dominate the products (~75% homologous). The remaining 25% products are reflective of multiple events, e.g. rearrangement and unrearranged product (46.3 kb, aneuploidy) or both unrearranged products (57.7 kb) indicative of intact dicentrics arising through BFB cycles.

## Discussion

Sequestering repair processes within sub-nuclear compartments and mobilization of DNA breaks to repair foci has been demonstrated for breaks within the nucleolus [25], pericentric heterochromatin [22] and constitutive heterochromatin [39,40]. The mechanisms for the spatial regulation are varied and likely depend upon a number of factors including cell cycle regulation of repair processes, protein compartmentalization, assembly, and rate of resection. The basis for the spatial and temporal segregation is often attributed to maintenance of repeats, e.g. rDNA in the nucleolus and alpha-satellite DNA in the centromere and constitutive heterochromatin. The pericentromeric chromatin in yeast is devoid of repeat DNA sequences, but shares physical attributes with distinct domains such as the nucleolus [27]. These attributes include enrichment in cohesin and condensin, density of DNA loops and proximity to tRNA genes [27]. We have used dicentric chromosome induced DSBs to reveal that repair pathways are differentially utilized for DNA breaks in the pericentromere vs. arms. Although budding yeast lack the genome complexity of larger eukaryotes, these results reveal unique biochemical aspects within the pericentromeric sub-domain.

Homology-based processes, HR or SSA, are more prevalent in cells containing dicentric chromosomes with two centromeres separated by > 40 kb relative to centromere separation < 20 kb (Fig 4A). In diploid cells containing dicentric chromosomes where the two centromeres were separated by 53, 72, and 120 kb, breaks could be physically mapped through PCR-based assays of single-nucleotide polymorphisms. About half of the breaks were broadly distributed between the two centromeres, while the remaining half were clustered within 10 kb from the conditional centromere (~50%) [11]. The dependence of repair events on Rad52 in the 46.3 and 57.7 kb dicentrics (< 10% viability, Fig 2 and as previously shown [7]) and Rad1 [12] were indicative of SSA as the primary repair pathway.

The reduced segregation fidelity of the dicentric chromosome on galactose [41], together with the fact that dicentric chromosomes can break and repair back to a dicentric, are indicative of multiple paths for cells to accumulate more than one copy of the dicentric chromosome. This is borne out in the analysis of repair products in cells with 46.3 and 57.7 kb dicentric chromosomes. The segment between eCEN3 and the second centromere at 46.3 or 57.7 kb contains an essential gene [42]. We therefore expected that an SSA event using 336 bp homology between the two centromeres (Fig 3A, right panel) would not be the only repair product following chromosome breakage and repair. Through PCR and whole genome sequencing, we found a circular monocentric derivative that contains the segment between the two centromeres (Fig 3A, left panel). Homology-based pathways that would give rise to these events are either a reciprocal cross over at the 336 bp of homology between eCEN3 and GALCEN3, or two independent SSA events. Homologous recombination is robust between sequences with 1 kb or greater homology. Below 1 kb, there is a drastic reduction in the efficiency of homologous recombination (decreased frequencies ranging from 20X [37,43] to 1,000X [44]). In addition, the major recombination event in mitosis is one-way gene conversion that is not associated with the reciprocal recombination product [45–47]. For independent SSA events, there would have to be a non-disjunction event that gives rise to cells with two dicentric chromosomes.

The dominant repair pathway at intermediate distances between centromeres (9.8 kb to 18.2 kb) was non-homologous end-joining. Chromosome breakage, followed by non-homologous end-joining, where either GALCEN3 or eCEN3 was deleted, resulted in a monocentric derivative chromosome (Figs 3 and 4). The deletions were all greater than 278 bp and can extend beyond several kb [10]. This is considerably larger than the deletions observed in canonical NHEJ (< 50 bp, [48]). One possibility is that the chromosomes undergo multiple cycles of BFB before stable monocentric deletions are attained. Alternatively, the breakage event severs DNA on either side of the centromere, due to the cell abscission mechanism of breakage. In this case, the entire centromere is removed, and the two ends are joined via end-joining. Interestingly, the smallest deletions of GALCEN3 we have observed are 230 bps (n = 189 [10] and Fig 5). This is approximately the size the nuclease resistant centromere core [35,36]. The breaks incurred at the centromere reveal a physiological significance to the regions of protein-free zones flanking centromere in vivo [35,36].

The propensity for end-joining within the pericentromere is not simply a consequence of cell cycle stage or lack of ability to utilize homology-based mechanisms. Upon deletion of *LIF1*, there is little to no change in viability, but almost a complete switch to homologous events, depending on the centromere distance (Fig 4B). Lif1 is a potent suppressor of homologous recombination due to its function in inhibiting 5’ resection [38,49]. It has been suggested that HR is suppressed in the centromere and nucleolus due to potential catastrophes upon DNA repeat expansion, loss, or translocation. That the pericentromere in yeast lacks repeats, but suppresses HR nonetheless, is indicative of alternative mechanisms responsible for local suppression of HR. The density of DNA loops in chromosome sub-domains may create an environment that allows local regulation of repair pathways.

Repair of breaks in the pericentromere are largely independent of Rad52 and Mrc1, unlike breaks along chromosome arms whose centromeres are separated by > 40 kb (Fig 2). Since DNA repair of dicentric chromosomes through end-joining is robust, the reliance of repair events on Rad52 and Mrc1 is indicative of regulatory mechanisms that shunt events toward a different pathway depending on the position of the breaks. The molecular signature of the initiating event for homology-based recombination at the centromere repeats is not clear. Since the breaks tend to cluster at either of the two centromeres [11], a break proximal to one of the two centromeres could be the initiating event. However due to the high viability and frequency of repair through RCO (Fig 2), it is unlikely that the initiating event is dependent on the site of chromosome breakage at the centromere. An alternative mechanism has been proposed in which the replication checkpoint promotes sister chromatid recombination at stalled forks [50,51]. Single-stranded DNA at the replication fork can be used to initiate recombination between sister chromatids [51]. In the case of dicentric chromosomes, this could lead to reciprocal exchange between the homologous centromeres independent of resection from the DSB break site. Since the efficiency of resection is reduced as the length between direct repeats increases [52], there is a strong kinetic prediction that repair of dicentric chromosomes with greater centromere-centromere distances will favor mechanisms that do not require resection.

The DNA replication machinery including fork protection complexes such as Mrc1/Tof1/Csm3 [30,31] prevent gross chromosomal rearrangements at centromeres by Rad52-dependent SSA [53] and formation of Rad52 foci following replication stress [50]. It has been proposed that the rate of the replicative helicase through centromere may be tightly regulated to minimize formation of ssDNA. There is a natural replication pause through the yeast centromere [31,54], that is dependent on Tof1 [31]. The ability to tightly couple DNA unwinding with synthesis may be particularly acute in centromeres. Delaying replication fork progression following DNA damage [30,55], may result in ssDNA gap formation at centromere, providing the means for exchange between sister centromeres, or homologous centromeres in the special case of dicentrics.

**Fig 6:**
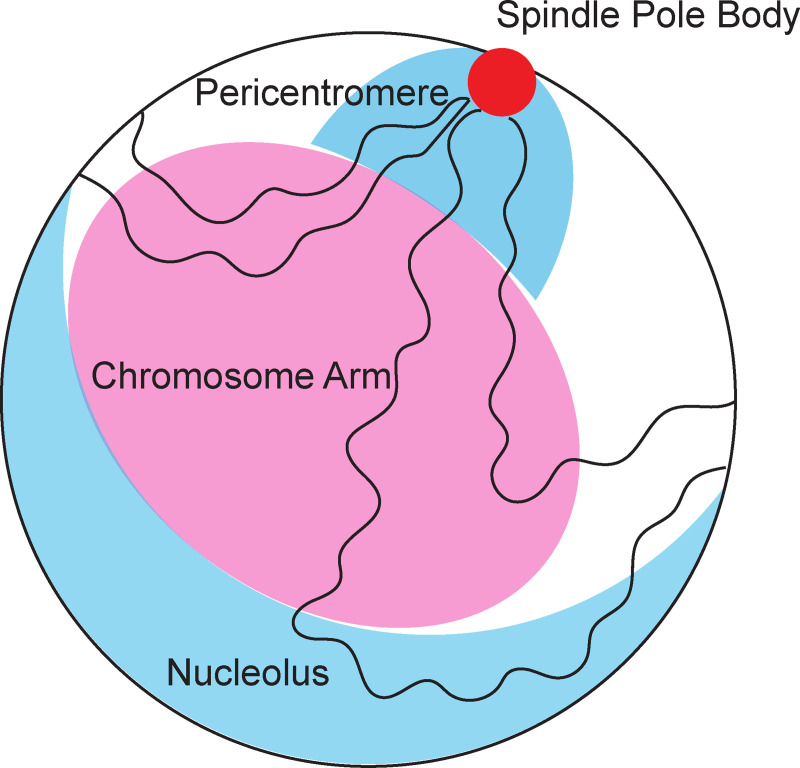
Illustration of sub-domains in the yeast nucleus. The major region of specialization in the nucleus is the nucleolus, where the rDNA (~1.5 Mbp) in chromosome XII resides. In budding yeast the nucleolus is typically on the other side of the nucleus from the spindle pole body (red circle). Chromosomes (4 of 16 shown) are organized in a Rabl configuration such that the 16 centromeres are tethered to the spindle pole body. Telomeres are organized into 4–6 clusters along the periphery of the nuclear envelope. The pericentromeres (defined as the centromere flanking DNA enriched in cohesin and condensin) are clustered as a consequence of centromere tethering. The pericentromere spans ~800 Kbp. We propose that the pericentromere is a second sub-domain of biochemical specialization.

There is precedence for the spatial control of repair pathways across phylogeny. An enzymatically-induced DSB is transiently relocated from the nucleolus to an extranucleolar site where HR repair proteins (Rad52) can be recruited in budding yeast [25]. Within the nucleolus in *Arabidopsis*, NHEJ is the favored mechanism responsible for maintaining the integrity of rDNA [56]. In human cells, knocking out components of the NHEJ pathway has a greater effect on viability in cells induced with DSBs in the nucleolus compared to the HR pathway [57]. Taken together, these results suggest that, similar to in the nucleolus, EJ is favored over HR as the dominant mechanism of repair within the pericentromere (Fig 6). It might be the case that HR proteins are excluded from the pericentromere as they seem to be from the nucleolus to ensure the fidelity of the centromere. In addition, it has been recently shown that mammalian centromeres harbor a unique repertoire of repair proteins [28,29]. Just as the rDNA is sequestered within the nucleolus, the centromere and the pericentromere may represent another form of sequestration due to the essential and unique requirements for chromosome segregation.

## Methods

### Materials and methods

#### Strain construction

One set of primers was used to lift the GALCEN3-HB or GALCEN-URA3 construct out of an existing strain or plasmid (pJB2#7) and has homology to the insertion site in the genome; another was used to screen for the correct insertion of the fragment after transformation. pJB2#7 contains a URA3 GAL1-CEN3 DNA fragment flanked by pBR322 vector DNA sequences inserted into the 5’end of the HIS4 gene at the position of the SalI restriction site. There are no repeated sequences in the construct.

The primers used to construct the 6.5 kb dicentric were Ldb16 up new (GCACATACACTTATGTGGTTCACCGTGCCGCTGCTGTGTTTATCTGTTGCTCGACATGTGCTGagatccagttcgatgtaacc) and Ldb16 bottom (TTTCTCACTATAAAAAAAGAAGAAATTACTTTAAATTGTTTGTCTATTCCAACATAATCATTAGcattaggaagcagcccagtagta), to screen, Ldb16 galcen chk up (CAACCAAGCGTATGTGCAACATTTT) and Ldb16 galcen chk dn (TGTTGACAATACTAGTGGAAGCACG). The primers used to construct the 9.8 kb dicentric were Ilv6 top (CTTAGAGAAGCCACCAAGGTATTGTGTCTTTAACCTTTTTCGTATCTGGCAAAATCGAAGagatccagttcgatgtaacc) and Ilv6 bottom (GTACGTTTGTACGAGGTGACGCGTTACTAAACTATTTTTTTCTTTTGGTTTTCTGCTTTCCcattaggaagcagcccagtagta), to screen, Ilv6 chk up (CTTCAAATCGTGTACCATCTGCTAC) and Ilv6 chk dn (ACTATTGCACCACCACACTTCCACC). The primers used to construct the 12.3 kb dicentric were Gbp2 top (ATCGCTGGAAGTGGTGCTCTTTTACAGGGATTAAATAAGGTTATTCTTTTTGGTCAAAATGagatccagttcgatgtaac) and Gbp2 bottom (GATAACGTATAAATAATAAGGAAGCGGGCGGGTTATAAATAACTTTAATAGTTATATTTATcattaggaagcagcccagtagta), to screen, Gbp2 chk up (GACATCATCCAAACCACCTCTTACG) and Gbp2 chk dn (ATTTTCAAGAGGATTCGGTTCTGTC). The primers used to construct the 18.2 kb dicentric were Dcc1 top (CTCCTAGAGATTTCGATCACCATCGTGGTGCTCTTTGTCATACGCATAGAATTGACAAAAagatccagttcgatgtaacc) and Dcc1 bottom (ACCCTAGGTCTTGGCAACTGGCAATCGCTCAACATGACCTAATTTATAGCTTAGGGTTCTcattaggaagcagcccagtagta), to screen, Dcc1 chk up (TCTTGGCGAATCCCACGACGTCCAT) and Dcc1 chk dn (GCGAGTTGGAAGATATATCGACGGA). The primers used to construct the 46.3 kb dicentric were US galcen Hyg New (ACCAAAGCAAGACATGGTCTCCAAGTGGCAAAATCCAACGTTTTCTTGTTCAACGATAAACagatccagttcgatgtaacc) and Downstream galcen Hyg (AATATGAGCGTTGTCTAGGGTTGGTGTATTCTTCGAAGAAATCTATAGCAAAGGCCATCGcattaggaagcagcccagtagta), to screen, Galcen 4080 (CTACCGTTAATTGATGATCTGG) and His4 Popout Up (TAGTATAAGATTCCTCTGGAGCGTC). The primers used to construct the 57.7 kb dicentric were 57kb dicentric insert US (AAGGAATTAATTTTAGCAATTTCCTAAAATTGTTGAGCGGCGCTATCAATGCCGCACAATAGATCCAGTTCGATGTAACC) and 57kb dicentric insert DS (TTGTCATCTAAGACCATGCTTTAGCTTCTGTCATGTATAATGCAACGTGTCTTAGATTATCATTAGGAAGCAGCCCAGTAGTA), to screen, 57kb dicentric check US (GTTTACACTACGCATCTTTCAATA) and 57kb dicentric check DS (GCCTATTTTACTCTAAGCAATTCAT). rad52::LEU2 mutants were generated by transformation with digested pSM20 plasmid. lif1::nat and lif1::his mutants were generated with pFA6-nat and pFA6-his plasmids and primers Lif1 3’ KO (CAGTTTTCGACGTGTCAATGCATAGAACTGAGAGAATTGTGCATTGGATGACTTATTTATGTAGGGTGCTcggatccccgggttaattaa) and Lif1 5’ KO (TCTCAAATGATGCGATACTATAATACTCTTTGCCATATATTACATTCATTCATAAATAGGgaattcgagctcgtttaaac) and screened with Lif1 chk up (CCGGAAGCGTATTTTGAAAAGCCAT) and Lif1 chk dn (GAGTTTTCGATCACTCACATAGAA). Mrc1::nat mutants were generated with the pFA6-nat plasmid and primers Mrc1 5’ up (CAGACAAACAACTAAGGAAGTTCGTTATTCGCTTTTGAACTTATCACCAAATATTTTAGTG cggatccccgggttaattaa) and Mrc1 3’ dn (TTTTTTAATGCGACTACTTCAAGACAGCTTCTGGAGTTCAATCAACTTCTTCGGAAAAGAgaattcgagctcgtttaaac) and screened with Mrc1 chk up new (TACTTCAAGACAGCTTCTGGAGTT) and Mrc1 chk dn new (ATAGAATGCGTAAAACGCGTTTGC). NFS1 was supplemented in the genome in the 46.3 kb dicentric by adding a Nat marker to the endogenous NFS1 gene in WT J1781D using the pFA6-nat plasmid and primers NFS1Upstream to add marker (TTTTGTGTGGTGCCCTCCTCCTTGTCAATATTAATGTTAAAGTGCAATTCTTTTTCCTTAcggatccccgggttaattaa) and NFS1Downstream to add marker (AGAAGGAGAAAAAGGAGGATGTAAAGGAATACAGGTAAGCAAATTGATACTAATGGCTCAgaattcgagctcgtttaaac), screened with primers NFS1chk up (AAATTAGGAATCATAGTTTCATGA) and NFS1 chk dn (TAGACGAAACTATATACGCAATCTA) for proper marker insertion, then the NFS1-nat construct was lifted out of the genome and transformed into the 46.3 kb dicentric strain with primers Upstream to lift nfs1 and marker going into Trp (tgagtcgtggcaagaataccaagagttcctcggtttgccagttattaaaagactcgtatt AAGACCGACAGAGTTCATTAGTGTGT) and Downstream from marker insert (ggaataaacgaatgaggtttctgtgaagctgcactgagtagtatgttgcagtcttttggaGTAAGCAAATTGATACTAATGGCTCA). Insertion was screened by primers insertion chk us (gattacggcattgatatcgtccaa) and insertion chk ds (aagttcacctgtcccacctgctt). The strains used for whole-genome sequencing were generated by inserting a Nat marker adjacent to eCEN3 using the plasmid pFA6-nat and primers CEN3 marker up (GTGAGCTCCGCCAATTGATTGTTTTGTTTTGAATATATATTGATGCTTAACAATTTAGTCG cggatccccgggttaattaa) and CEN3 marker dn (TTTACGTAGAACTCCCACATGGGGAGTAAATTGAAAAAAAGCCTGTAAGAGGTAGGTTTT gaattcgagctcgtttaaac) and screened using primers CEN3 marker chk up (GGTAGCATCGTACTAACTATTGGC) and CEN3 marker chk dn (TCCAAGGGCAGTAGAACTTAAGAT) in the 46.3 kb dicentric strain. All strains used in this study are listed in S1 Table.

#### Yeast media

Cells were grown on liquid and solid Yeast Bacto-Peptone Glucose (YPD) and Yeast Bacto-Peptone Galactose (YPG) media.

#### Viability assays

Cell viability was determined by growing strains in YPG, diluting in sterile water, and spreading with sterile glass beads onto both YPD and YPG plates. Plates were incubated at 24°C. After incubation, single colonies were counted, and percent viability was calculated by dividing the number of colonies on YPD by the number of colonies on YPG.

#### Dicentric time course assay

50mL cell cultures in YPG were incubated at 24°C until logarithmic growth was reached (optical density between 0.4–0.6 at OD_660_). Cultures were pelleted and resuspended in 50 mL YPD, then incubated at 24°C in an orbital shaker. Fresh cultures were inoculated every 24 hours. After 72 hours of growth on in YPD, cells were plated as outlined above. Plates were incubated at 24°C for 3–4 days. Singles colonies were picked into 2mL YPD cultures using sterile toothpicks and incubated at 24°C in an orbital shaker for 24 hours. DNA was extracted using a phenol-chloroform method.

#### Polymerase chain reaction (PCR)

Primers for GALCEN3-CEN3 recombination were GC1 (TCGACTACGCGATCATGGCG) and eC2 (GGGTGGGAAACTGAAGAAATC); reciprocal product, eC1 (TCAATAGCTTGCAGCGTAGCTAA) and GC2 (CACGATGCGTCCGGCGTAGA); GALCEN3, GC1 and GC2; CEN3, eC1 and eC2 (See Fig 1). All PCR reactions were carried out using 25uL reactions with GoTAQ Green (Promega, Madison, WI) and 1uL of 80–100 ng/uL DNA. DNA concentration was measured using a Qubit fluorometer (Invitrogen, Waltham, MA). The standard PCR protocol throughout was 98°C for 2 min followed by 30 cycles of 95°C for 1 min, 52°C for 30 sec, 68°C for 3 min; then 68°C for 5 min and held at 4°C. S4 Fig PCR reactions were carried out with a slightly different protocol: 98°C for 2 min followed by 30 cycles of 95°C for 1 min, 52°C for 1 min, 68°C for 5 min; then 68°C for 5 min and held at 4°C.

#### Gel Electrophoresis and Analysis

5–10 uL of each PCR product was run on a 1% agarose gel alongside a comparable volume of GeneRuler 1 kb Plus DNA Ladder (Thermo Scientific, Waltham, MA) at constant amperage (200 mA). Gels were stained in 0.5 ug/mL ethidium bromide and imaged using a ChemiDoc imaging system (Biorad, Hercules, CA).

#### GALCEN3 sequencing

GALCEN3 sequences (Fig 5) were determined by performing PCR on DNA obtained from single colonies from 72hr time course YPD dilution plates. For 9.8 kb, 12.3 kb, and 18.2 kb dicentric strains, primers GC1 and GC2 were used to amplify GALCEN3 fragments. For the 57.7 kb strain, primers GC1 and 57 kb chk dn (GCCTATTTTACTCTAAGCAATTCAT) were used. The resulting bands were then run on a gel. Bands were extracted and purified using the GeneJET Gel Extraction Kit (Thermo Scientific). DNA was sequenced by Eton Bioscience. Sequences were analyzed using SnapGene v5.0.4.

#### Whole genome sequencing

Cells were grown up in YPG, then plated onto YPD and YPG. Colonies that survived on YPD plates were grown in 2 mL YPD liquid cultures at 24°C. DNA was extracted using a phenol-chloroform method. After the DNA was extracted from the cells, Qiagen QiaSeqFX DNA Library Kits were used to prep the DNA for short read full genome sequencing at the UNC-CH High Throughput Sequencing Facility using a Hi-Seq Sequencer. The results of the sequencing were received and uploaded to the UNC Longleaf computing cluster, where an alignment program was used to align the sequences to the reference genome and compile the genome library. The genome was visualized using the Integrative Genomics Viewer (IGV), created by the Broad Institute and by the University of California at San Diego [58,59]. Using IGV, the aligned genome showed that no genetic information was gained or lost. An additional control was the Gal 1 promoter sequence used to control the GALCEN3 construct. When viewed using IGV, the sequence at Gal 1 had two times the number of reads as compared to other sequences that were not repeated, indicating that the number of reads was a reliable measure for number of reads taken from the reaction.

## Supporting information

S1 Fig Supplementation of NFS1 does not affect viability or distribution of repair products in the 46.3 kb Dicentric. (A) Quantitative analysis of cell viability (single colony growth on glucose/galactose) of WT and trp1::NFS1 46.3 kb dicentric strains. Integration of *NFS1* in the genome at the *TRP1* locus did not significantly affect the viability of the strain; Student’s T-test p-value is 0.30198757. Error bars indicate ± SEM. (B) Chart showing distribution of repair events after 72 hours in the 46.3 kb dicentric with and without integration of the *NFS1* gene elsewhere in the genome. Cultures were grown on glucose for 72 hours, then plated on YPglucose. 30 of the resulting single colonies were analyzed by PCR that probed for CEN3 (eC1-eC2), GALCEN3 (GC1-GC2), rearrangement product (GC1-eC2), and reciprocal product (eC1-GC2), see Fig 4 for detailed category descriptions. Integration of *NFS1* in the genome at the *TRP1* locus did not significantly affect the distribution of repair events.
(EPS)

S2 Fig Reciprocal Circle from 46.3 kb Dicentric Strain. (A) Schematic depicting the circular product following dicentric breakage in a chromosome with centromeres 46.3 kb apart. The circle contains a functional centromere, ARS307 and ARS308 origins of replication, and DNA from the position of GALCEN3 at 68,000 to CEN3 at 114,300. Primers were used to amplify overlapping fragments of the circle, as indicated by colored arcs. Unique enzymes were used to verify the identity of each PCR product. Primers are listed in S3 Table. (B) Agarose gel containing both uncut and cut PCR products used to verify the circular product. The first 9 products are shown. (C) Sizes of cut and uncut fragments from panel B.
(EPS)

S3 Fig Growth Curves of Monocentric Derivatives. Cells were identified to contain the various monocentric derivatives through PCR analysis as described in the text. Single colonies were grown to logarithmic growth phase and at 2 hour intervals, aliquots were taken and the optical density of the culture was determined. The growth curves were repeated three times for each sample, error bars indicated ± SEM. There was no difference in growth rates for cells experiencing HR or EJ (for either CEN3 deletions or GALCEN deletions). There was a major difference in the growth rate of cells experiencing SSA and loss of information between the endogenous centromere and the GALCEN 6.5 kb toward the left arm. Student’s t-test values comparing 6.5 kb SSA to 46.3 kb EJ GALCEN3Δ can be found in S4 Table. Hours 4, 6, and 8 are significantly different, p<0.5.
(EPS)

S4 Fig Reciprocal Circle from 12.3 kb Dicentric Strain. (A) Schematic depicting the circular product following dicentric breakage in a chromosome with centromeres 12.3 kb apart. The circle contains a functional centromere, ARS307 and ARS308 origins of replication, and DNA from the position of GALCEN3 at 112,000 to CEN3 at 114,300. There are no essential genes present of the circle, but it is stably maintained. Primers were used to amplify overlapping fragments of the circle, as indicated by colored arcs. Unique enzymes were used to verify the DNA sequence of each PCR product. Primers are listed in S5 Table. (B) Agarose gel containing both uncut and cut PCR products used to verify the circular product. Arrows indicate cut fragments of expected size. Set A uncut size is 4.5 kb, cut sizes are 3.2 kb and 1.3 kb; Set B uncut 4.5 kb, cut 2.6 kb and 1.9 kb; Set C uncut 4.5 kb, cut 2.4 kb and 2.1 kb.
(EPS)

S5 Fig Reciprocal Circle from 9.8 kb Dicentric Strain. (A) Schematic depicting the circular product following dicentric breakage in a chromosome with centromeres 9.8 kb apart. The circle contains a functional centromere, ARS307 and ARS308 origins of replication, and DNA from the position of GALCEN3 at 104,456 to CEN3 at 114,300. There are no essential genes present on the circle, but it is stably maintained. Primers were used to amplify overlapping fragments of the circle, as indicated by colored arcs. Unique enzymes were used to verify the DNA sequence of each PCR product. Primers are listed in S6 Table. (B) Agarose gel containing both uncut and cut PCR products used to verify the circular product. Arrows indicate cut fragments of expected size. Set A uncut size is 3.5 kb, cut sizes are 2.2 kb and 1.3 kb; Set B uncut 2.8 kb, cut 1.4 kb and 1.4 kb; Set C uncut 3.4 kb, cut 1.2 kb and 2.2 kb; Set D uncut 1.5 kb, cut 1 kb and 0.5 kb.
(EPS)

S1 Table Strains. Summary of strains used in this study.
(DOCX)

S2 Table Student’s T-Test p-values for Fig 2. Comparing each mutant to WT for each dicentric distance.
(DOCX)

S3 Table Primers Used to Map 46.3 kb Reciprocal Circle. Primers correspond to S2 Fig.
(DOCX)

S4 Table Student’s T-test p-values for S3 Fig. Comparing 6.5 kb SSA to 46.3 kb EJ.
(DOCX)

S5 Table Primers Used to Map 12.3 kb Reciprocal Circle. Primers correspond to S4 Fig.
(DOCX)

S6 Table Primers Used to Map 9.8 kb Reciprocal Circle. Primers correspond to S5 Fig.
(DOCX)
