# Supplementary material for: Behavior of dicentric chromosomes in budding yeast
Source: PLoS Genet. 2021 Mar 18;17(3):e1009442. doi: 10.1371/journal.pgen.1009442 (PMC8009378; doi:10.1371/journal.pgen.1009442)
Supplement: S1 Table — Summary of strains used in this study. (DOCX) [file pgen.1009442.s006.docx]

**S1 Table. Strains.** Summary of strains used in this study.

| Strain | Genotype |
| --- | --- |
| J1781D | MATa ade1 met14 ura3-52 leu2-3,112 his3-11,15 |
| DCY1232.1 | J1781D GALCEN3-HB::ldb16 |
| SLY2.1 | J1781D GALCEN3-HB::ilv6 |
| SLY6.1 | J1781D GALCEN3-HB::gbp2 |
| SLY1.1 | J1781D GALCEN3-HB::dcc1 |
| DCY1214.1 | J1781D GALCEN3-HB::his4 |
| DCY1355.1 | J1781D GALCEN3-URA3::sro9 |
| DCY1238.1 | J1781D GALCEN3-HB::ldb16 rad52::LEU2 |
| SLY8.1 | J1781D GALCEN3-HB::ilv6 rad52::LEU2 |
| SLY11.1 | J1781D GALCEN3-HB::gbp2 rad52::LEU2 |
| SLY9.1 | J1781D GALCEN3-HB::dcc1 rad52::LEU2 |
| DCY1227.1 | J1781D GALCEN3-HB::his4 rad52::LEU2 |
| DCY1368.1 | J1781D GALCEN3-URA3::sro9 rad52::LEU2 |
| DCY1299.1 | J1781D GALCEN3-HB::ldb16 lif1::nat |
| DCY1297.1 | J1781D GALCEN3-HB::ilv6 lif1::nat |
| DCY1298.1 | J1781D GALCEN3-HB::gbp2 lif1::nat |
| DCY1296.1 | J1781D GALCEN3-HB::dcc1 lif1::nat |
| DCY1300.1 | J1781D GALCEN3-HB::his4 lif1::nat |
| DCY1367.1 | J1781D GALCEN3-URA3::sro9 lif1::HIS3 |
| DCY1310.0 | J1781D GALCEN3-HB::ldb16 mrc1::nat |
| DCY1318.1 | J1781D GALCEN3-HB::ilv6 mrc1::nat |
| DCY1319.1 | J1781D GALCEN3-HB::gbp2 mrc1::nat |
| DCY1312.1 | J1781D GALCEN3-HB::dcc1 mrc1::nat |
| DCY1131.1 | J1781D GALCEN3-HB::his4 mrc1::nat |
| DCY1369.1 | J1781D GALCEN3-URA3::sro9 mrc1::nat |
| DCY1113.1 | J1781D GALCEN3-URA3::his4 CEN3:nat rad52::LEU2 |
| DCY1096.1 | J1781D GALCEN3-URA3::his4 CEN3:nat |
| DCY1360.1 | J1781D GALCEN3-URA3::his4 trp1::NFS1 |
| DCY1214.1.01 | J1781D GALCEN3-HB::his4 monocentric derivative (lost GALCEN3 and CEN3) |
| DCY1214.1.10 | J1781D GALCEN3-HB::his4 monocentric derivative (lost GALCEN3) |
| SLY2.1.03 | J1781D GALCEN3-HB::ilv6 (lost GALCEN3) |
| SLY2.1.18 | J1781D GALCEN3-HB::ilv6 (lost CEN3) |
| DCY1232.1.01 | J1781D GALCEN3-HB::ldb16 (SSA, loss of DNA between CEN3 and GALCEN3) |
