# Supplementary material for: Behavior of dicentric chromosomes in budding yeast
Source: PLoS Genet. 2021 Mar 18;17(3):e1009442. doi: 10.1371/journal.pgen.1009442 (PMC8009378; doi:10.1371/journal.pgen.1009442)
Supplement: S2 Table — Comparing each mutant to WT for each dicentric distance. (DOCX) [file pgen.1009442.s007.docx]

**S2 Table. Student’s T-Test p-values for Fig 2.** Comparing each mutant to WT for each dicentric distance.

| WT strain | *lif1Δ* | *mrc1Δ* | *rad52Δ* |
| --- | --- | --- | --- |
| 6.5 kb | 0.145881668 | 0.116188944 | 2.58E-05 |
| 9.8 kb | 0.172956115 | 3.45502E-05 | 5.74E-12 |
| 12.3 kb | 0.078032554 | 0.343924208 | 0.000137 |
| 18.2 kb | 0.000351014 | 0.608192891 | 0.000101 |
| 46.3 kb | 0.257109511 | 7.06835E-10 | 4.81E-05 |
| 57.7 kb | 0.079152558 | 2.56652E-15 | 2.03021E-16 |
