# Supplementary material for: Behavior of dicentric chromosomes in budding yeast
Source: PLoS Genet. 2021 Mar 18;17(3):e1009442. doi: 10.1371/journal.pgen.1009442 (PMC8009378; doi:10.1371/journal.pgen.1009442)
Supplement: S3 Table — Primers correspond to S2 Fig. (DOCX) [file pgen.1009442.s008.docx]

**S3 Table. Primers Used to Map 46.3 kb Reciprocal Circle.** Primers correspond to S2 Fig.

| Primer Name | Sequence (5’-3’) |
| --- | --- |
| eC1 | TCAATAGCTTGCAGCGTAGCTAA |
| Gbp2 Away Reverse Complement | AATCACGTCTAGCATAAGAGATC |
| Ilv6 Reciprocal Bottom | AAGACCTACTATTGCACCACCAC |
| Ilv6 Away Reverse Complement | ATAGGCTCATGGTTATAGATACG |
| Gbp2 Set 3 Bottom | CATATTGCGGCTAGTTATATACC |
| Gbp2 Set 2 Top | CAAAGCTTGATCTTCTCCATAAT |
| LDB Outward Bottom | CACCATTGGCTTTATAATCTGAT |
| LDB Inward Top | GCCACTTTATTATCGTCTTTAGT |
| LDB Inward Bottom | AGTATGGACATTATAAACGTGTG |
| LDB Outward Top | CTTCCATTTATAAATGGAGAGCT |
| Cen to Tel Reverse | TTAGCTACGCTGCAAGCTATTGA |
| 40kb Recip Bik1 | TAAACATGGCATGGCGATCAGC |
| Bik1 top strand | GTATTCTTTACTGAGCGAATCGT |
| Rnq1 Bottom | GTTGTTGGAATTCATGAAAGATG |
| Rnq1 Top | AAACGTATAGCAAAGATCTGAAA |
| Fus1 Bottom | TATTCGGTGGTTTAGTATACAGG |
| Fus1 Top | TTAAGAGCAGGATATAAGCCATC |
| Ars306 Bottom | CATCAATAACGAACAGCACTATT |
| Hbn1 Top Strand | GTAAATAGTACGACGAGCAGTTA |
| Agp1 up Reverse | TAGCATACGTGTAATGATAGACG |
| Agp1 up | CGTCTATCATTACACGTATGCTA |
| Agp1 dn | GGTCTCATCGAGTCATAGATAAG |
| Agp1 dn Reverse | CTTATCTATGACTCGATGAGACC |
| Kcc4 Bottom | TCTTATTGCGGTTCTTCTTATTC |
| Kcc4 Top | AATACCTATCTATATCCACTGGC |
| YCLW delta 15 Reverse Complement | TTATTGGAACAGTTGAGTTGAGTT |
| YCLW delta 15 | AACTCAACTCAACTGTTCCAATAA |
| G 85.7 kb Bottom | GGCTGTAATAATGAAGATTATACATGG |
| Sup53 Bottom Reverse Complement | ATCAATTGTCCTGTACTTCCT |
| Leu2 End Bottom | CTAAATCACCAGTTCTGATACCT |
| Leu2 End Top | AGGTATCAGAACTGGTGATTTAG |
| Nfs1 Bottom | GAAGACTAAGAAACACATCATCAC |
| Nfs1 Top | GCATCTTCCAATTCCTTCAAATC |
| Dcc1 Away | CCTCATTGAAGATCAAAGCCTTT |
| Dcc1 Away Reverse Complement | AAAGGCTTTGATCTTCAATGAG |
| Bud 3.2 Bottom | AGAGGATAATTTACATACCATTGAGAA |
| Bud 2.2 Top | AAGTTACTACACTTCAGATGGAT |
| Bud3 Bottom | ATGTAGATGCATCTTCATTAGTT |
| Bud3 Top | TTATTCCTAAGCATTACCGTAAT |
| Gbp2 Away | GATCTCTTATGCTAGACGTGATT |
