# Supplementary material for: Behavior of dicentric chromosomes in budding yeast
Source: PLoS Genet. 2021 Mar 18;17(3):e1009442. doi: 10.1371/journal.pgen.1009442 (PMC8009378; doi:10.1371/journal.pgen.1009442)
Supplement: S4 Table — Comparing 6.5 kb SSA to 46.3 kb EJ. (DOCX) [file pgen.1009442.s009.docx]

**S4 Table. Student’s T-test p-values for S3 Fig.** Comparing 6.5 kb SSA to 46.3 kb EJ.

| 0 hr | 0.744445 |
| --- | --- |
| 2 hr | 0.360342 |
| 4 hr | 0.023269 |
| 6 hr | 0.000645 |
| 8 hr | 0.010567 |
