# Supplementary material for: Behavior of dicentric chromosomes in budding yeast
Source: PLoS Genet. 2021 Mar 18;17(3):e1009442. doi: 10.1371/journal.pgen.1009442 (PMC8009378; doi:10.1371/journal.pgen.1009442)
Supplement: S5 Table — Primers correspond to S4 Fig. (DOCX) [file pgen.1009442.s010.docx]

**S5 Table. Primers Used to Map 12.3 kb Reciprocal Circle.** Primers correspond to S4 Fig.

| Primer Name | Sequence (5’-3’) |
| --- | --- |
| GBP_Set_1 Top | ACTATTTGGATAAAGTCACTCCT |
| GBP_Set_1 Bottom | AATGTAATCGGAAAGAACTACTG |
| GBP_Set_2 Top | CAAAGCTTGATCTTCTCCATAAT |
| GBP_Set_2 Bottom | TCATCTTGCTGTAAGGACATATC |
| GBP_Set_3 Top | TGCCATGAGAAGATCTTATGATA |
| GBP_Set_3_Bottom | CATATTGCGGCTAGTTATATACC |
