# Supplementary material for: Behavior of dicentric chromosomes in budding yeast
Source: PLoS Genet. 2021 Mar 18;17(3):e1009442. doi: 10.1371/journal.pgen.1009442 (PMC8009378; doi:10.1371/journal.pgen.1009442)
Supplement: S6 Table — Primers correspond to S5 Fig. (DOCX) [file pgen.1009442.s011.docx]

**S6 Table. Primers Used to Map 9.8 kb Reciprocal Circle.** Primers correspond to S5 Fig.

| Primer Name | Sequence (5’-3’) |
| --- | --- |
| eC1 | TCAATAGCTTGCAGCGTAGCTAA |
| Ilv6 Outward Bottom | CTAGTAATATGGAGACACATCGT |
| Ilv6 Inward Top | ATGTGTCATAATCTCTTCTCTCC |
| Ldb16 Outward Bottom | CACCATTGGCTTTATAATCTGAT |
| Ldb16 Inward Top | GCCACTTTATTATCGTCTTTAGT |
| Ldb16 Inward Bottom | AGTATGGACATTATAAACGTGTG |
| Ldb16 Outward Top | CTTCCATTTATAAATGGAGAGCT |
| CentoTel Reverse | TTAGCTACGCTGCAAGCTATTGA |
